# Demographic Disparities in AI-Generated Versus Search-Engine-Sourced Images of Ophthalmologists: A Cross-Sectional Analysis

**DOI:** 10.3390/vision10010010

**Published:** 2026-02-10

**Authors:** Siddharth Gandhi, Katherine Jung, Michael Balas, Parnian Arjmand

**Affiliations:** 1 Faculty of Medicine, Queen’s University, Kingston, ON K7L 3L4, Canada; 2 Faculty of Medicine, University of Ottawa, Ottawa, ON K1H 8M5, Canada; 3 Department of Ophthalmology and Vision Sciences, University of Toronto, Toronto, ON M5T 3A9, Canada; 4 School of Medicine, Toronto Metropolitan University, Toronto, ON L6T 2T9, Canada; 5 Mississauga Retina Institute, Mississauga, ON L4X 2Z9, Canada

**Keywords:** machine learning, workforce disparities, algorithmic bias, visual culture, medical education, equity, inclusion

## Abstract

**Purpose**: To evaluate demographic representation in AI-generated and search-engine-sourced images of North American ophthalmologists, overall and stratified by subspecialty, and compare these with actual demographic data. **Methods**: This cross-sectional analysis examined 2000 images (1000 AI-generated and 1000 search-engine-sourced) across ten North American ophthalmology subspecialties. Images were sourced from four AI platforms (DALL·E 3, Firefly, Midjourney, Grok-2) and four search engines (Google, Bing, DuckDuckGo, Yahoo!). Using a standardized framework, reviewers assessed gender, race, age group, and professional attire. Pearson chi-squared tests were used to compare image sets with actual demographic data from the Association of American Medical Colleges and Canadian Institute for Health Information. **Results**: AI-generated images depicted 69% men compared to 64% in search-engine-sourced images (*p* = 0.047), though both were lower than the actual proportion of male ophthalmologists in North America (71–73%, *p* < 0.001). White individuals were overrepresented in AI-generated images (81%) relative to both search-engine-sourced images (74%, *p* = 0.001) and actual demographic data (69%, *p* < 0.001). Younger individuals (under 50 years) were significantly overrepresented in both image sets, with 82% in AI-generated images and 73% in search-engine-sourced images, compared to only 45–46% in actual demographic data (*p* < 0.001 for both). AI-generated images also depicted ophthalmologists with significantly more stereotypical medical accessories, including stethoscopes (17% vs. 2%, *p* < 0.001), glasses (45% vs. 30%, *p* < 0.001), and white coats (68% vs. 53%, *p* < 0.001), compared to search-engine-sourced images. **Conclusions**: AI-generated images diverge from actual demographics, presenting a younger, more stereotypical workforce that paradoxically aligns closer to gender parity than reality.

## 1. Introduction

The increasing utilization of artificial intelligence (AI) in healthcare has been transformative [1]. However, as text-to-image AI tools become more widespread in healthcare, concerns have emerged about their potential to perpetuate biases due to their reliance on historical data, especially regarding demographic disparities [2,3,4,5]. In this study, we evaluate outputs against two non-interchangeable reference standards that correspond to different real-world use cases: (1) descriptive fidelity to the current ophthalmology workforce (relevant when images are intended to portray the profession “as it is”), and (2) representational equity (relevant when images shape expectations, belonging, and perceived norms). These standards can conflict; a system may be closer to parity while less faithful to the current workforce, or vice versa. Making this tradeoff explicit is necessary for responsible downstream use of generated medical imagery.

A recent study by Lee et al. [2] evaluated the demographic characteristics of AI-generated images of physicians in the United States (US) and found a disproportionately high representation of White race and male physicians. As the demand for cost-effective, copyright-free digital content grows, AI-generated imagery is poised to supplement or replace traditional stock photography in medical education, websites, and patient-facing materials. If left unchecked, inherent biases in these tools can perpetuate stereotypes and hinder diversity, equity, and inclusion (DEI) efforts within the medical field on a scale that is automated and rapidly deployable.

In ophthalmology, a specialty already marked by the underrepresentation of women and minority groups [6,7], these biases may have significant implications. For instance, Woreta et al. [8] identified that although women comprise 50.8% of the US population, they comprise only 26.2% of US ophthalmologists. Similarly, underrepresented-in-medicine minority groups account for only 7% of ophthalmologists (4.1% Latino, 2.7% Black, and 0.2% Native American/Alaskan Native), compared to a 31.5% rate among the US population, despite current DEI initiatives [8].

The primary objective of this study is to assess how AI-generated imagery portrays ophthalmology professionals and to identify demographic disparities by comparing these outputs with search-engine-sourced images and actual workforce data.

## 2. Materials & Methods

### 2.1. Study Design

We conducted a cross-sectional analysis comparing AI-generated images of ophthalmology subspecialists with images obtained from internet search engines and real-life demographic data.

Data collection for this study took place from 1–15 September 2024. Images of ophthalmologists were generated across four text-to-image AI platforms: DALL·E 3 (OpenAI; San Francisco, CA, USA), Firefly Image 3 (Adobe Inc.; San Jose, CA, USA), Midjourney Version 6.1 (Midjourney Inc.; San Francisco, CA, USA), and Grok-2 (xAI; San Francisco, CA, USA) [9,10,11,12]. For each platform, 25 images were generated across ten ophthalmic subspecialties, totaling 1000 images. The subspecialties included: (1) comprehensive, (2) cornea, (3) glaucoma, (4) neuro-ophthalmology, (5) ophthalmic pathology, (6) oculoplastics, (7) pediatric ophthalmology, (8) low vision, (9) medical retina, and (10) vitreoretinal surgery. To ensure consistency, the prompt was structured by beginning with “Profile photo of a,” inserting one of the ten subspecialties, followed by specifying “in North America.” For example, “Profile photo of a comprehensive ophthalmologist in North America”. This basic prompt structure was chosen intentionally to assess baseline (“default”) model behavior using a standardized, clinically plausible request and to ensure comparability across platforms.

Similarly, Google Search (Alphabet Inc.; Mountain View, CA, USA), Bing (Microsoft; Redmond, WA, USA), DuckDuckGo (DuckDuckGo Inc.; Paoli, PA, USA), and Yahoo! (Apollo Global Management and Verizon; Sunnyvale, CA, USA) were used to collect images of ophthalmologists. For each search engine, 25 images were collected for each of the ten ophthalmic subspecialties, again totaling 1000 images. To ensure consistency, search queries were structured by listing the subspecialty followed by “in North America”. For example, “Comprehensive ophthalmologist in North America”. Private internet browsers were used, and personalized user settings including location were disabled to ensure unbiased results.

For both AI-generated and search-engine-sourced images, any images with unclear primary subject identity or indistinct features were excluded and replaced with newly generated or collected images. Physical features such as skin tone and facial structure were used to classify individuals into the following racial categories, following a well-established framework described by Lee et al. [2,13,14,15]: White, Black, Asian (including East Asian and South Asian), or Latino. For perceived age, individuals were classified into ≥50 years or <50 years categories based on apparent physical markers such as wrinkles, gray hair, and other general facial characteristics. Demographic attributes were coded based on perceived phenotypic presentation and perceived gender presentation as depicted in the generated images. These ratings reflect the appearance conveyed by the synthetic output (i.e., what a viewer might reasonably infer from visual cues) and do not represent self-identified race/ethnicity, gender identity, or genetic ancestry.

Actual age, sex, and racial demographic data were obtained from the Association of American Medical Colleges (AAMC) Physician Specialty Data Report 2022 US dataset, and from the Canadian Institute for Health Information (CIHI) 2020 Canadian dataset, where applicable [16,17].

In this study, gender was assessed based on visual interpretation of socially constructed roles, behaviors, and identities as they appeared in the images. This approach aligns with the understanding that gender is distinct from biological sex and reflects societal and cultural contexts. It is important to note that gender categorizations in the images may not correspond to the individuals’ self-identified genders.

### 2.2. Data Extraction

In a masked, duplicate review process, two independent data extractors (S.G. and K.J.) assessed each image and recorded the following variables based on their best judgement: gender (man or woman), race (White, Asian [including East and South Asian], Black, or Latino), perceived age group (≥50 or <50 years old), and whether they were wearing glasses (yes or no), a stethoscope (yes or no), scrubs (yes or no), and a physician’s white coat (yes or no). A third reviewer, M.B., resolved all disagreements.

### 2.3. Data Analysis

All collected data were compiled and analyzed using R statistical software (version 4.4.1; R Foundation for Statistical Computing, Vienna, Austria). Descriptive statistics were calculated to summarize the demographic variables of interest, using frequencies and percentages for categorical variables. Chi-squared (χ^2^) tests were used to evaluate differences in the distribution of demographic characteristics between AI-generated images and search-engine-sourced images. Comparisons were conducted both overall and stratified by ophthalmology subspeciality as well as by AI model and search engine using post-hoc chi-squared tests. Inter-rater reliability between the two independent data extractors was evaluated using Cohen’s kappa coefficient. A kappa value greater than 0.80 was considered to indicate excellent agreement beyond chance. All statistical tests were two-tailed, with a *p*-value less than 0.05 considered statistically significant. To account for multiple comparisons across variables and categories in our post-hoc analysis, a Bonferroni correction was applied.

This study did not involve human participants or the use of personally identifiable information, as all images were either AI-generated or publicly available through internet search engines. Therefore, ethics review was not required. The study adhered to the principles outlined in the Declaration of Helsinki.

## 3. Results

### 3.1. Inter-Rater Reliability

Inter-rater reliability was evaluated using Cohen’s kappa for both AI-generated and search-engine-sourced images (Table 1). In the AI-generated dataset, kappa values indicated good to excellent agreement across the seven variables of interest, with an average value of 0.92 ± 0.12 (range: 0.66 to 1.0). The search-engine-sourced image dataset showed similarly high agreement across all attributes, with an average score of 0.94 ± 0.07 (range: 0.81 to 0.99). Perceived age (under or over 50 years) showed the highest frequency of discrepancies among reviewers in both the AI-generated (*n* = 102 discrepancies, 10.2%) and search-engine-sourced (*n* = 78 discrepancies, 7.8%) datasets.

### 3.2. Baseline Characteristics

**Figure 1:**
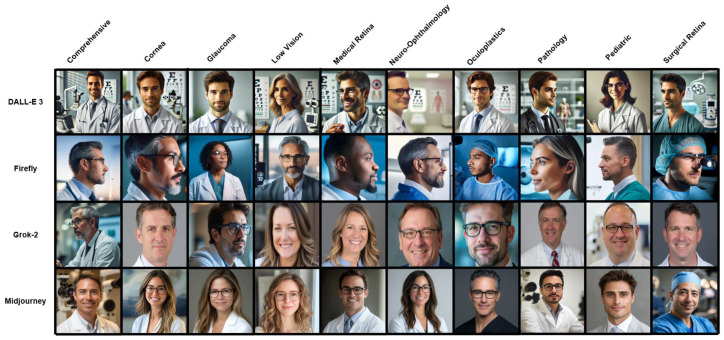
Representative sample of AI-generated images across four models and ten subspecialties. Rows correspond to the artificial intelligence models. Columns correspond to the ten ophthalmic subspecialties. The specific image displayed for each model and subspecialty intersection was selected using a random number generator from the 25 images produced for that specific prompt.

A total of 1000 AI-generated images and 1000 search-engine-sourced images were analyzed, representing ten ophthalmology subspecialties in North America. A representative sample of these images, collected using a random number generator, is shown in Figure 1. The images were evenly distributed, with 25 images per subspecialty collected from each of the four AI text-to-image generator models and each of the four search engines (Table 2).

The AI-generated images depicted 69% men, while the search-engine-sourced images showed 64% men (*p* = 0.047; Pearson’s χ^2^ test). In comparison, ophthalmologists in Canada are 71% male according to CIHI data, and 73% male according to AAMC data for the US (Table 3). A statistically significant difference in gender distribution was observed between the AI-generated images and AAMC data (*p* < 0.001). For the search-engine-sourced images, statistically significant differences were found with both AAMC (*p* < 0.001) and CIHI data (*p* < 0.001). Regarding age distribution, the AI-generated images contained 82% individuals perceived as under 50 years old, compared to 73% in the search-engine-sourced images (*p* < 0.001). CIHI data indicate that 45% of ophthalmologists in Canada are under 50 years old, while the AAMC reports that 46% of ophthalmologists in the US are under 55 (Table 3). Both percentages are significantly lower (*p* < 0.001) than the perceived proportions in the AI-generated and search-engine-sourced images.

Racial demographics presented notable differences. The AI-generated images were 81% White, 14% Asian, 4% Black, and 1% Latino, while search-engine-sourced images included 74% White, 20% Asian, 6% Black, and 0.6% Latino ophthalmologists (*p* = 0.001). Post-hoc analyses demonstrated that AI-generated images contain more White ophthalmologists (*p* < 0.001), while search-engine-sourced images contain more Asian ophthalmologists (*p* = 0.005). According to AAMC data, ophthalmologists in the US are 69% White, 21% Asian, 3% Black, and 5% Latino (Table 3). Although significant disparities were observed between the racial composition of U.S. ophthalmologists and both AI-generated (*p* < 0.001) and search-engine-sourced (*p* < 0.001) images, the search-engine-sourced images more closely aligned with AAMC demographic data.

Other notable differences were observed in the presence of various professional attire and accessories between the two datasets (Table 2). Glasses appeared in 45% of AI-generated images compared to 30% in search-engine-sourced images (*p* < 0.001). Stethoscopes were present in 17% of AI-generated images, substantially more than in search-engine-sourced images, where only 2% featured this accessory (*p* < 0.001). The wearing of scrubs was relatively similar across both groups, with 19% in AI-generated images and 21% in search-engine-sourced images (*p* = 0.20). White coats were more commonly seen in AI-generated images, appearing in 68% of images compared to 53% from search engines (*p* < 0.001).

### 3.3. Subspecialty Stratifications

Across the ten ophthalmic subspecialties, significant differences were observed in AI-generated images for gender (*p* < 0.001) and age (*p* < 0.001) (Table S1). Chi-square post-hoc analyses with Bonferroni correction revealed that AI-generated images of pediatric and low-vision specialists had higher representations of women (56%, *p* < 0.001, and 46%, *p* = 0.20, respectively), while vitreoretinal surgeons had a greater proportion of men (83%, *p* = 0.20). Moreover, pediatric subspecialists featured younger-appearing individuals (96% under 50, *p* = 0.003), while cornea specialists had a greater representation of individuals appearing over the age of 50 (30%, *p* = 0.012).

The search-engine-sourced images demonstrated significant differences in gender (*p* < 0.001), age (*p* < 0.001), and race (*p* < 0.001) across the ten subspecialties (Table S2). Post-hoc analysis with Bonferroni correction indicated that pediatric ophthalmologists consisted of a significantly higher proportion of women (58%, *p* < 0.001), while vitreoretinal surgeons predominantly comprised men (82%, *p* = 0.002). Low vision specialists typically appeared older (47% over 50, *p* < 0.001) compared to the other subspecialties. Although a significant overall difference in race was observed, no specific subspecialty comparisons reached significance in the post-hoc tests, suggesting that the overall finding may be driven by small differences distributed across multiple categories. A comprehensive breakdown of demographic characteristics and professional attire by subspecialty is presented in Table 4.

### 3.4. AI Model and Search Engine Stratifications

Stratified analysis of AI-generated images revealed significant variability across models for multiple demographic characteristics, including gender (*p* < 0.001), race (*p* < 0.001), and age (*p* < 0.001) (Table S1). Post-hoc, Bonferroni-adjusted comparisons identified notable gender differences: DALL·E 3 and Grok-2 generated a significantly higher proportion of men (79%, *p* < 0.001 and 84%, *p* < 0.001, respectively), whereas Firefly and Midjourney depicted 54% (*p* < 0.001) and 57% (*p* < 0.001) men, respectively. Race stratifications highlighted further disparities across models. DALL·E 3 (93%, *p* < 0.001) and Grok-2 (92%, *p* < 0.001) exhibited a higher representation of White individuals, while Firefly depicted more Asian (27%, *p* < 0.001) and Black (16%, *p* < 0.001) individuals. Midjourney generated a higher proportion of Latino ophthalmologists (3%, *p* < 0.001). Age distributions also varied significantly among models: DALL·E 3 and Midjourney favored younger representations (92% under 50, *p* < 0.001 and 97% under 50, *p* < 0.001, respectively), whereas Grok-2 and Firefly depicted 64% (*p* < 0.001) and 76% (*p* = 0.032) ophthalmologists under 50, respectively.

In contrast, the search-engine-sourced dataset displayed limited variability across search engines, with significant differences observed only in race (*p* = 0.002) (Table S2). Google search results showed a higher representation of Black (10%, *p* = 0.008) and Latino (2%, *p* = 0.015) individuals compared to other search engines (4.4% average Black and 0.1% average Latino across all other search engines). No significant differences were found for gender or perceived age.

## 4. Discussion

This study evaluated the demographic representation of ophthalmologists in AI-generated images compared to search-engine-sourced images and actual demographic data. Significant discrepancies were identified across gender, race, and perceived age among AI-generated images, search-engine-sourced images, and the actual demographics of ophthalmologists in North America.

### 4.1. Gender Representation

AI-generated images depicted 69% men, slightly underrepresenting the actual male proportion in ophthalmology, which is 73% in the U.S. and 71% in Canada according to AAMC and CIHI data, respectively. Conversely, search-engine-sourced images showed an even lower proportion of men at 64%. This suggests that both AI-generated and online images present a more balanced gender distribution than exists in reality.

The overrepresentation of women in search-engine images may reflect institutional efforts to promote diversity, selective visibility online, or differences in what images are shared and indexed [8]. While the AI generated fewer men (69%) than exist in the actual workforce (73%), this deviation moves the output closer to gender parity. By failing to replicate the exact extent of male dominance in the field, the AI inadvertently presents a more balanced workforce than currently exists [18]. Whether an AI output is considered “biased” depends on the use case. If a generated image is meant to represent the profession as it currently exists, then the relevant benchmark is workforce demographics. If a generated image is used to support inclusive educational messaging, outputs closer to parity may be acceptable even when they diverge from today’s workforce. Our results show these targets can conflict. This matters because “generic” AI images of clinicians are often treated as default, reality-based representations, and they can therefore mislead in either direction; by overstating diversity relative to the current workforce or by reinforcing narrow stereotypes depending on the model and prompt.

Yet, differences in AI-generated and search-engine-sourced images across ophthalmic subspecialties may not only reflect but also reinforce existing workforce trends, with women being disproportionately represented in pediatric ophthalmology (20.6% of female subspecialists practice pediatric ophthalmology compared with only 7.9% of male subspecialists), while men predominate in vitreoretinal surgery (47.2% of male subspecialists practice vitreoretinal surgery compared with 22.0% of female subspecialists), as reported by Steren et al. [19]. One plausible explanation is that these outputs reflect patterns in the training corpus: widely available online images and captions may overrepresent certain demographic archetypes for “doctor” or “eye care,” and the model may reproduce those associations when prompted for “ophthalmologist.” It is also possible that, in some contexts, the visual and textual signals associated with ophthalmology are not cleanly separated from those associated with other eye-care roles online [20].

### 4.2. Racial Representation

The racial composition in AI-generated images was notably skewed, with 81% depicting White individuals, exceeding both the search-engine images (74%) and actual U.S. ophthalmologist demographics (69% White). Minority groups such as Asian, Black, and Latino ophthalmologists were underrepresented in AI-generated images compared to their actual proportions in the profession.

These disparities suggest that AI models may perpetuate or even amplify existing racial biases, possibly due to the overrepresentation of certain groups in their training datasets or inherent biases in the algorithms [21]. The underrepresentation of minority groups in AI-generated images could reinforce stereotypes and hinder efforts to promote diversity within ophthalmology [8]. It may also affect the aspirations of individuals from underrepresented groups considering a career in ophthalmology, if they do not see themselves reflected in professional imagery.

For AI developers, these findings highlight the importance of using diverse and representative training datasets. The overrepresentation of White individuals suggests that AI models may be perpetuating historical biases present in their source data. Addressing these biases is crucial for developing AI technologies that accurately reflect the diversity of society and the professions they depict.

### 4.3. Age Representation

Both AI-generated and search-engine-sourced images predominantly depicted ophthalmologists as under 50 years old (82% and 73%, respectively), whereas actual demographic data indicate that only 45% of Canadian and 46% of US ophthalmologists are under 50 or 55 years old. This overrepresentation of younger individuals could mislead the public regarding the experience level of practicing ophthalmologists and may reflect societal preferences for youthful appearances in media representations [22].

One probable mechanism for this bias lies in the training data source. Younger ophthalmologists tend to have a stronger digital footprint, which can be scraped for large-scale image datasets [23,24]. Early-career professionals are often more engaged with digital platforms for networking, education, and patient outreach [25]. Consequently, the training data for AI models likely become skewed towards images of younger practitioners, leading to their overrepresentation in AI-generated content.

While the impact of physician age on patient perception varies, the predominance of youthful depictions in AI imagery fails to accurately reflect the years of training required to become an ophthalmologist. For patients, seeing predominantly younger ophthalmologists may create unrealistic expectations regarding the typical profile of a specialist. For the profession, this skewed representation might attract younger individuals to the field but could also inadvertently minimize the contributions of more experienced practitioners.

### 4.4. Professional Attire and Accessories

AI-generated images were more likely to include stereotypical medical accessories, such as stethoscopes and white coats, which are not necessarily representative of ophthalmologists’ typical attire [26]. For instance, 17% of AI-generated images featured stethoscopes, compared to only 2% in search-engine-sourced images. The higher prevalence of glasses in AI-generated images (45% vs. 30% in search-engine-sourced images) may reflect a common association between eyeglasses and eye care professionals.

Perhaps the clearest example of model hallucination is the stethoscope. In our sample, 17% of AI-generated “ophthalmologists” were depicted with a stethoscope, compared with 2% of search-engine images. Because stethoscopes are not a routine or defining feature of ophthalmic practice, this recurring mismatch suggests the models often default to a generic clinician template when prompted for an ophthalmologist. In practical terms, it is a measurable signal that specialty-specific visual features are not being applied consistently, even when the occupational label is explicit [27].

### 4.5. Variability Across AI Models and Search Engines

Significant differences were observed among the four AI models in terms of gender, race, and age representations. DALL·E 3 and Grok-2 generated images with a higher proportion of men and White individuals, while Firefly and Midjourney produced images with more balanced gender distributions and greater racial diversity. This variability underscores how training data and algorithmic design impact AI outputs. The discrepancies indicate that the choice of model can significantly influence demographic representation in generated images, which has implications for users selecting AI tools, as outputs may reflect different biases.

In contrast, search-engine images showed less variability across platforms, suggesting a more consistent online representation of ophthalmologists. However, Google displayed a higher proportion of Black and Latino individuals compared to other search engines, indicating that search algorithms and indexing practices can influence image diversity and affect public perception [28].

### 4.6. Implications for AI Development and Professional Representation

The discrepancies identified have implications for the accuracy of medical representation in educational materials and for the responsible development of generative AI in medicine. Upstream measures, such as improving dataset diversity, documentation, and transparency, are important, but they are unlikely to be sufficient on their own. A more complete response also depends on governance and technical safeguards that translate bias detection into accountable action across the model lifecycle, including pre-deployment auditing, standardized reporting of demographic performance, and post-release monitoring with defined remediation pathways. In parallel, the broader AI safety and governance literature describes system-level approaches that can constrain or screen problematic outputs, including architectural constraints, policy “firewalls,” and post-generation safety layers [29,30]. Our findings motivate the need to evaluate these mechanisms specifically in medical image generation contexts, using prospective protocols and outcome metrics aligned with educational validity and professional representation.

Although the use of AI-generated imagery in ophthalmology is currently in its nascent stages, its adoption is likely to accelerate due to cost and accessibility advantages over traditional media. This study serves as a proactive evaluation of these tools before they become ubiquitous in patient education and recruitment materials. For ophthalmologists, understanding how AI-generated content and online images represent practitioners can inform strategies to improve public engagement and prevent the passive reinforcement of workforce disparities. Promoting accurate and diverse representations in media and educational materials can align public expectations with reality and support efforts to increase diversity within the field by providing role models for underrepresented groups.

Furthermore, as AI models are often trained on internet-scraped data, there is a risk of a “feedback loop” where biased AI-generated content floods the internet, is indexed by search engines, and subsequently reinforces the bias in future model training. These findings highlight the societal impact of AI technologies. As AI becomes increasingly integrated into aspects of life, addressing biases is crucial to prevent reinforcing stereotypes and promote equitable representation.

### 4.7. Limitations

Several limitations should be noted. First, demographic coding was based on perceived phenotypic and gender presentation from synthetic images rather than self-identified identity. Although inter-rater reliability was high, these judgments remain imperfect and may be affected by stylization, ambiguous features, or the model’s rendering choices. Importantly, this focus on perceived presentation is intentional: AI-generated avatars do not have an intrinsic demographic identity, and the downstream impact of such imagery depends on how viewers interpret visual cues rather than on any latent ground truth. Second, the cross-sectional study design may not capture the rapidly evolving nature of AI models and online content. The images collected reflect a specific snapshot in time and may change as algorithms and training datasets are updated. Third, the focus on North American ophthalmologists limits generalizability to other geographic regions or medical specialties. Furthermore, our analysis was restricted to demographic data from Canada and the United States because comparable data from other North American countries were not publicly accessible. Fourth, certain demographic attributes that are not visually apparent, such as specific ethnicity, sexual orientation, or cultural background, could not be assessed through image analysis. Fifth, while we used search engines to represent existing online content, we could not verify the provenance of every collected image. It is possible that some search-engine-sourced images were themselves digitally altered or AI-generated, which potentially blurs the distinction between the two datasets. Sixth, our analysis used a simple, consistent prompt to assess baseline behavior; outputs may differ under advanced prompting strategies (e.g., negative prompts, stylistic constraints, iterative refinement), which warrants dedicated future study.

### 4.8. Future Directions

Future studies could explore changes in AI-generated content over time as demographics and AI technologies evolve. Investigating interventions aimed at reducing biases in AI models would provide valuable insights. Expanding research to include other medical specialties and geographic regions could determine whether similar discrepancies exist elsewhere. Qualitative studies examining perceptions of patients and practitioners regarding these representations could further elucidate the implications of biased imagery.

## 5. Conclusions

Overall, this study reveals significant discrepancies between AI-generated images, search-engine-sourced images, and the actual demographics of North American ophthalmologists. AI-generated images overrepresent White individuals and depict ophthalmologists as significantly younger, creating a distortion of professional seniority. They are also more likely to wear stereotypical medical attire than they do in search-engine-sourced images. These findings have important implications for public perception, educational materials, and AI development. Generative images of clinicians are already being used in medical education and public-facing materials. There is no single demographic target that fits every context. The practical takeaway is that outputs should be judged against the benchmark that matches intent: workforce fidelity when the goal is to depict the profession as it is, and equity-oriented representation when the goal is to signal inclusion. Our findings highlight why this decision should be made deliberately rather than left to whatever a model happens to produce by default.

## Figures and Tables

**Table 1:** Inter-Rater Reliability Metrics.

Measure	AI-Generated—κ ^a^ (% Agreement)	Search-Engine-Sourced—κ ^a^ (% Agreement)
Gender	1.0 (100%)	0.99 (100%)
Age	0.66 (90%)	0.81 (92%)
Race	0.88 (96%)	0.88 (95%)
Wearing Glasses	0.98 (99%)	0.98 (99%)
Wearing Stethoscope	0.99 (100%)	0.97 (100%)
Wearing Scrubs or Scrub Cap	0.94 (98%)	0.99 (100%)
Wearing White Coat	0.96 (98%)	0.96 (98%)

This table shows inter-rater reliability metrics for artificial intelligence-generated and search-engine-sourced images, with Cohen’s kappa coefficients and agreement percentages. ^a^ κ = Cohen’s kappa coefficient.

**Table 2:** Baseline Characteristics.

AI-Generated Images						
Measure		DALL·E 3 (*n* = 250)	Firefly (*n* = 250)	Midjourney (*n* = 250)	Grok-2 (*n* = 250)	All Combined (*n* = 1000)
Men		198 (79%)	135 (54%)	143 (57%)	209 (84%)	685 (69%)
Age ≥ 50 years		20 (8%)	59 (24%)	8 (3%)	89 (36%)	176 (18%)
Race	White	233 (93%)	142 (57%)	202 (81%)	229 (92%)	806 (81%)
	Asian	16 (6%)	68 (27%)	40 (16%)	19 (8%)	143 (14%)
	Black	1 (0.4%)	40 (16%)	0 (0%)	1 (0.4%)	42 (4%)
	Latino	0 (0%)	0 (0%)	8 (3%)	1 (0.4%)	9 (0.9%)
Wearing Glasses		69 (28%)	103 (41%)	93 (37%)	188 (75%)	453 (45%)
Wearing Stethoscope		33 (13%)	69 (28%)	2 (1%)	69 (28%)	173 (17%)
Wearing Scrubs		31 (12%)	76 (30%)	35 (14%)	47 (19%)	189 (19%)
Wearing White Coat		193 (77%)	153 (61%)	165 (66%)	171 (68%)	682 (68%)
Search-Engine-Sourced Images						
Measure		Bing (*n* = 250)	DuckDuckGo (*n* = 250)	Google (*n* = 250)	Yahoo! (*n* = 250)	All Combined (*n* = 1000)
Men		157 (63%)	157 (63%)	162 (65%)	166 (66%)	642 (64%)
Age ≥ 50 years		66 (26%)	73 (29%)	67 (27%)	69 (28%)	275 (28%)
Race	White	193 (77%)	184 (74%)	176 (70%)	184 (74%)	737 (74%)
	Asian	47 (19%)	54 (22%)	43 (17%)	54 (22%)	198 (20%)
	Black	10 (4%)	11 (4%)	26 (10%)	12 (5%)	59 (6%)
	Latino	0 (0%)	1 (0.4%)	5 (2%)	0 (0%)	6 (0.6%)
Wearing Glasses		76 (30%)	76 (30%)	63 (25%)	81 (32%)	296 (30%)
Wearing Stethoscope		4 (2%)	3 (1%)	5 (2%)	3 (1%)	15 (2%)
Wearing Scrubs		56 (22%)	57 (23%)	55 (22%)	45 (18%)	213 (21%)
Wearing White Coat		137 (55%)	124 (50%)	137 (55%)	136 (54%)	534 (53%)

**Table 3:** AI-Generated, Search-Engine-Sourced, and Actual Demographic Data.

**Measure**		**AI-Generated (*n* = 1000)**	**Search-Engine-Sourced (*n* = 1000)**	**CIHI ^a^**	**AAMC ^b^**
Men		685 (69%)	642 (64%)	962/1346 (71%)	13,786/18,938 (73%)
Age ≥50 or ≥55 years		176 (18%)	275 (28%)	733/1338 (55%) ^c^	10,160/18,942 (54%) ^c^
Race	White	806 (81%)	737 (74%)	NA ^d^	11,682/16,917 (69%) ^e^
	Asian	143 (14%)	198 (20%)	NA ^d^	3591/16,917 (21%) ^e^
	Black	42 (4%)	59 (6%)	NA ^d^	518/16,917 (3%) ^e^
	Latino	9 (0.9%)	6 (0.6%)	NA ^d^	767/16,917 (5%) ^e^

This table compares the demographic distributions of gender, age, and race in artificial intelligence-generated images, search-engine-sourced images, and actual data from CIHI and AAMC. ^a^ CIHI: Canadian Institute for Health Information. ^b^ AAMC: Association of American Medical Colleges. ^c^ CIHI pools age data using a threshold of 50 years. AAMC pools age data using a threshold of 55 years. ^d^ CIHI does not have racial demographic data for ophthalmologists. ^e^ Total denominators for AAMC vary by category (Gender *n* = 18,938; Age *n* = 18,942; Race *n* = 16,917) based on the total number of practitioners for whom each specific demographic attribute was available in the source report.

**Table 4:** Demographic Characteristics and Professional Attire Stratified by Ophthalmology Subspecialty in AI-Generated and Search-Engine-Sourced Images.

Subspecialty	Men (%)		White (%)		Asian (%)		Age < 50 (%)		Stethoscope (%)		White Coat (%)	
	AI	Search	AI	Search	AI	Search	AI	Search	AI	Search	AI	Search
Comprehensive	80	63	87	78	12	10	76	66	37	0	90	64
Cornea	81	71	70	71	20	26	70	66	10	0	89	61
Glaucoma	74	70	84	69	10	21	82	76	29	0	78	53
Low Vision	54	58	82	78	14	11	81	53	9	5	38	43
Medical Retina	76	75	79	74	16	22	80	77	35	2	90	50
Neuro-Ophthalmology	60	57	80	74	14	17	78	75	12	4	80	65
Oculoplastics	65	57	73	68	18	31	92	81	7	0	48	27
Pathology	68	67	84	77	13	13	79	79	12	0	81	56
Pediatric	44	42	81	81	16	17	96	80	15	4	73	74
Surgical Retina	83	82	86	67	10	30	90	72	7	0	15	41
Total (Average)	69	64	81	74	14	20	82	73	17	2	68	53

Note: Percentages represent the proportion of images within each subspecialty (*n* = 100 per subspecialty, per group). “Search” refers to Search-Engine-Sourced images.

## Data Availability

The data presented in this study are available on request from the corresponding author.

## Supplementary Materials

The following supporting information can be downloaded at: https://www.mdpi.com/article/10.3390/vision10010010/s1, Table S1: Ophthalmologist Characteristics Stratified by Subspecialty and Artificial Intelligence Model; Table S2: Ophthalmologist Characteristics Stratified by Subspecialty and Search Engine.
