# Supplementary material for: Demographic Disparities in AI-Generated Versus Search-Engine-Sourced Images of Ophthalmologists: A Cross-Sectional Analysis
Source: Vision (Basel). 2026 Feb 10;10(1):10. doi: 10.3390/vision10010010 (PMC12921965; doi:10.3390/vision10010010)
Supplement: Supplementary file 1 [file vision-10-00010-s001.zip › vision-4112415-supplementary.pdf]

Table S1: Ophthalmologist Characteristics Stratified by Subspecialty and Artificial Intelligence Model
Table S2: Ophthalmologist Characteristics Stratified by Subspecialty and Search Engine

**Table S1:** Ophthalmologist Characteristics Stratified by Subspecialty and Artificial Intelligence Model							
**Subspecialty**	**Measure**		**All Models Combined (n = 1000)**	**DALL·E 3 (n = 250)**	**Firefly (n = 250)**	**Midjourney (n = 250)**	**Grok-2 (n = 250)**
All Subspecialties Combined (n = 1000)	Men		685 (69%)	198 (79%)	135 (54%)	143 (57%)	209 (84%)
	Age ≥50 years		176 (18%)	20 (8%)	59 (24%)	8 (3%)	89 (36%)
	Race	White	806 (81%)	233 (93%)	142 (57%)	202 (81%)	229 (92%)
		Asian	143 (14%)	16 (6%)	68 (27%)	40 (16%)	19 (8%)
		Black	42 (4%)	1 (0.4%)	40 (16%)	0 (0%)	1 (0.4%)
		Latino	9 (0.9%)	0 (0%)	0 (0%)	8 (3%)	1 (0.4%)
	Wearing Glasses		453 (45%)	69 (28%)	103 (41%)	93 (37%)	188 (75%)
	Wearing Stethoscope		173 (17%)	33 (13%)	69 (28%)	2 (1%)	69 (28%)
	Wearing Scrubs		189 (19%)	31 (12%)	76 (30%)	35 (14%)	47 (19%)
	Wearing White Coat		682 (68%)	193 (77%)	153 (61%)	165 (66%)	171 (68%)
Comprehensive Ophthalmology (n = 100)	Men		80 (80%)	25 (100%)	13 (52%)	20 (80%)	22 (88%)
	Age ≥50 years		24 (24%)	4 (16%)	6 (24%)	1 (4%)	13 (52%)
	Race	White	87 (87%)	25 (100%)	17 (68%)	21 (84%)	24 (96%)
		Asian	12 (12%)	0 (0%)	7 (28%)	4 (16%)	1 (4%)
		Black	1 (1%)	0 (0%)	1 (4%)	0 (0%)	0 (0%)
		Latino	0 (0%)	0 (0%)	0 (0%)	0 (0%)	0 (0%)
	Wearing Glasses		56 (56%)	9 (36%)	14 (56%)	8 (32%)	25 (100%)
	Wearing Stethoscope		37 (37%)	8 (32%)	17 (68%)	0 (0%)	12 (48%)
	Wearing Scrubs		7 (7%)	1 (4%)	2 (8%)	0 (0%)	4 (16%)
	Wearing White Coat		90 (90%)	23 (92%)	24 (96%)	23 (92%)	20 (80%)
Cornea and External Disease (n = 100)	Men		81 (81%)	18 (72%)	20 (80%)	19 (76%)	24 (96%)
	Age ≥50 years		30 (30%)	0 (0%)	17 (68%)	2 (8%)	11 (44%)
	Race	White	70 (70%)	22 (88%)	9 (36%)	16 (64%)	23 (92%)
		Asian	20 (20%)	3 (12%)	8 (32%)	7 (28%)	2 (8%)
		Black	8 (8%)	0 (0%)	8 (32%)	0 (0%)	0 (0%)
		Latino	2 (2%)	0 (0%)	0 (0%)	2 (8%)	0 (0%)
	Wearing Glasses		45 (45%)	2 (8%)	15 (60%)	10 (40%)	18 (72%)
	Wearing Stethoscope		10 (10%)	0 (0%)	10 (40%)	0 (0%)	0 (0%)
	Wearing Scrubs		5 (5%)	2 (8%)	2 (8%)	0 (0%)	1 (4%)
	Wearing White Coat		89 (89%)	25 (100%)	23 (92%)	23 (92%)	18 (72%)
Glaucoma (n = 100)	Men		74 (74%)	22 (88%)	12 (48%)	17 (68%)	23 (92%)
	Age ≥50 years		18 (18%)	2 (8%)	6 (24%)	1 (4%)	9 (36%)
	Race	White	84 (84%)	23 (92%)	18 (72%)	18 (72%)	25 (100%)
		Asian	10 (10%)	1 (4%)	5 (20%)	4 (16%)	0 (0%)
		Black	3 (3%)	1 (4%)	2 (8%)	0 (0%)	0 (0%)
		Latino	3 (3%)	0 (0%)	0 (0%)	3 (12%)	0 (0%)
	Wearing Glasses		47 (47%)	2 (8%)	19 (76%)	10 (40%)	16 (64%)
	Wearing Stethoscope		29 (29%)	7 (28%)	8 (32%)	1 (4%)	13 (52%)
	Wearing Scrubs		10 (10%)	3 (12%)	5 (20%)	1 (4%)	1 (4%)
	Wearing White Coat		78 (78%)	13 (52%)	17 (68%)	25 (100%)	23 (92%)
Neuro-Ophthalmology (n = 100)	Men		60 (60%)	19 (76%)	10 (40%)	7 (28%)	24 (96%)
	Age ≥50 years		22 (22%)	3 (12%)	5 (20%)	1 (4%)	13 (52%)
	Race	White	80 (80%)	23 (92%)	15 (60%)	22 (88%)	20 (80%)
		Asian	14 (14%)	2 (8%)	5 (20%)	2 (8%)	5 (20%)
		Black	5 (5%)	0 (0%)	5 (20%)	0 (0%)	0 (0%)
		Latino	1 (1%)	0 (0%)	0 (0%)	1 (4%)	0 (0%)
	Wearing Glasses		46 (46%)	12 (48%)	6 (24%)	6 (24%)	22 (88%)
	Wearing Stethoscope		12 (12%)	1 (4%)	9 (36%)	0 (0%)	2 (8%)
	Wearing Scrubs		3 (3%)	0 (0%)	3 (12%)	0 (0%)	0 (0%)

Table S1: Ophthalmologist Characteristics Stratified by Subspecialty and Artificial Intelligence Model

	Wearing White Coat		80 (80%)	23 (92%)	18 (72%)	22 (88%)	17 (68%)
Ophthalmic Pathology (n = 100)	Men		68 (68%)	20 (80%)	8 (32%)	18 (72%)	22 (88%)
	Age ≥50 years		21 (21%)	6 (24%)	4 (16%)	0 (0%)	11 (44%)
	Race	White	84 (84%)	22 (88%)	17 (68%)	22 (88%)	23 (92%)
		Asian	13 (13%)	3 (12%)	6 (24%)	2 (8%)	2 (8%)
		Black	2 (2%)	0 (0%)	2 (8%)	0 (0%)	0 (0%)
		Latino	1 (1%)	0 (0%)	0 (0%)	1 (4%)	0 (0%)
	Wearing Glasses		52 (52%)	7 (28%)	10 (40%)	14 (56%)	21 (84%)
	Wearing Stethoscope		12 (12%)	3 (12%)	5 (20%)	0 (0%)	4 (16%)
	Wearing Scrubs		5 (5%)	2 (8%)	1 (4%)	1 (4%)	1 (4%)
	Wearing White Coat		81 (81%)	15 (60%)	24 (96%)	23 (92%)	19 (76%)
Ophthalmic Plastic Surgery (n = 100)	Men		65 (65%)	16 (64%)	13 (52%)	15 (60%)	21 (84%)
	Age ≥50 years		8 (8%)	0 (0%)	0 (0%)	1 (4%)	7 (28%)
	Race	White	73 (73%)	24 (96%)	6 (24%)	19 (76%)	24 (96%)
		Asian	18 (18%)	1 (4%)	11 (44%)	5 (20%)	1 (4%)
		Black	8 (8%)	0 (0%)	8 (32%)	0 (0%)	0 (0%)
		Latino	1 (1%)	0 (0%)	0 (0%)	1 (4%)	0 (0%)
	Wearing Glasses		33 (33%)	7 (28%)	0 (0%)	4 (16%)	22 (88%)
	Wearing Stethoscope		7 (7%)	2 (8%)	1 (4%)	0 (0%)	4 (16%)
	Wearing Scrubs		38 (38%)	2 (8%)	25 (100%)	6 (24%)	5 (20%)
	Wearing White Coat		48 (48%)	24 (96%)	1 (4%)	11 (44%)	12 (48%)
Pediatric Ophthalmology (n = 100)	Men		44 (44%)	11 (44%)	15 (60%)	3 (12%)	15 (60%)
	Age ≥50 years		4 (4%)	0 (0%)	1 (4%)	0 (0%)	3 (12%)
	Race	White	81 (81%)	21 (84%)	15 (60%)	25 (100%)	20 (80%)
		Asian	16 (16%)	4 (16%)	7 (28%)	0 (0%)	5 (20%)
		Black	3 (3%)	0 (0%)	3 (12%)	0 (0%)	0 (0%)
		Latino	0 (0%)	0 (0%)	0 (0%)	0 (0%)	0 (0%)
	Wearing Glasses		41 (41%)	11 (44%)	11 (44%)	5 (20%)	14 (56%)
	Wearing Stethoscope		15 (15%)	2 (8%)	7 (28%)	0 (0%)	6 (24%)
	Wearing Scrubs		14 (14%)	0 (0%)	7 (28%)	0 (0%)	7 (28%)
	Wearing White Coat		73 (73%)	24 (96%)	18 (72%)	14 (56%)	17 (68%)
Low-Vision (n = 100)	Men		54 (54%)	21 (84%)	17 (68%)	5 (20%)	11 (44%)
	Age ≥50 years		19 (19%)	4 (16%)	9 (36%)	1 (4%)	5 (20%)
	Race	White	82 (82%)	24 (96%)	13 (52%)	21 (84%)	24 (96%)
		Asian	14 (14%)	1 (4%)	9 (36%)	4 (16%)	0 (0%)
		Black	4 (4%)	0 (0%)	3 (12%)	0 (0%)	1 (4%)
		Latino	0 (0%)	0 (0%)	0 (0%)	0 (0%)	0 (0%)
	Wearing Glasses		69 (69%)	9 (36%)	20 (80%)	19 (76%)	21 (84%)
	Wearing Stethoscope		9 (9%)	1 (4%)	0 (0%)	0 (0%)	8 (32%)
	Wearing Scrubs		7 (7%)	0 (0%)	2 (8%)	0 (0%)	5 (20%)
	Wearing White Coat		38 (38%)	12 (48%)	7 (28%)	1 (4%)	18 (72%)
Medical Retina (n = 100)	Men		76 (76%)	21 (84%)	16 (64%)	16 (64%)	23 (92%)
	Age ≥50 years		20 (20%)	1 (4%)	10 (40%)	0 (0%)	9 (36%)
	Race	White	79 (79%)	25 (100%)	13 (52%)	18 (72%)	23 (92%)
		Asian	16 (16%)	0 (0%)	8 (32%)	7 (28%)	1 (4%)
		Black	4 (4%)	0 (0%)	4 (16%)	0 (0%)	0 (0%)
		Latino	1 (1%)	0 (0%)	0 (0%)	0 (0%)	1 (4%)
	Wearing Glasses		40 (40%)	6 (24%)	6 (24%)	11 (44%)	17 (68%)
	Wearing Stethoscope		35 (35%)	8 (32%)	12 (48%)	1 (4%)	14 (56%)
	Wearing Scrubs		9 (9%)	0 (0%)	4 (16%)	2 (8%)	3 (12%)
	Wearing White Coat		90 (90%)	25 (100%)	21 (84%)	22 (88%)	22 (88%)
	Men		83 (83%)	25 (100%)	11 (44%)	23 (92%)	24 (96%)

Table S1: Ophthalmologist Characteristics Stratified by Subspecialty and Artificial Intelligence Model

Vitreoretinal Surgery (n = 100)	Age ≥50 years		10 (10%)	0 (0%)	1 (4%)	1 (4%)	8 (32%)
	Race	White	86 (86%)	24 (96%)	19 (76%)	20 (80%)	23 (92%)
		Asian	10 (10%)	1 (4%)	2 (8%)	5 (20%)	2 (8%)
		Black	4 (4%)	0 (0%)	4 (16%)	0 (0%)	0 (0%)
		Latino	0 (0%)	0 (0%)	0 (0%)	0 (0%)	0 (0%)
	Wearing Glasses		24 (24%)	4 (16%)	2 (8%)	6 (24%)	12 (48%)
	Wearing Stethoscope		7 (7%)	1 (4%)	0 (0%)	0 (0%)	6 (24%)
	Wearing Scrubs		91 (91%)	21 (84%)	25 (100%)	25 (100%)	20 (80%)
	Wearing White Coat		15 (15%)	9 (36%)	0 (0%)	1 (4%)	5 (20%)

This table presents the demographic characteristics by ophthalmic subspecialty and artificial intelligence model. Data are presented as frequency n (%). The “All Models Combined” column represents the aggregate data across all four AI platforms (total n = 1000; n = 100 per subspecialty). The individual model columns (DALL·E 3, Firefly, Midjourney, Grok-2) represent the data specific to that platform (total n = 250; n = 25 per subspecialty). Within each subspecialty, each model contributes n = 25 images (total n = 100 per subspecialty across all four models). Percentages are calculated based on the number of images within that specific model and subspecialty intersection (i.e., within-cell denominator n = 25).

**Table S2:** Ophthalmologist Characteristics Stratified by Subspecialty and Search Engine							
**Subspecialty**	**Measure**		**All Engines Combined (n = 1000)**	**Bing (n = 250)**	**DuckDuckGo (n = 250)**	**Google (n = 250)**	**Yahoo! (n = 250)**
All Subspecialties Combined (n = 1000)	Men		642 (64%)	157 (63%)	157 (63%)	162 (65%)	166 (66%)
	Age ≥50 years		275 (28%)	66 (26%)	73 (29%)	67 (27%)	69 (28%)
	Race	White	737 (74%)	193 (77%)	184 (74%)	176 (70%)	184 (74%)
		Asian	198 (20%)	47 (19%)	54 (22%)	43 (17%)	54 (22%)
		Black	59 (6%)	10 (4%)	11 (4%)	26 (10%)	12 (5%)
		Latino	6 (0.6%)	0 (0%)	1 (0.4%)	5 (2%)	0 (0%)
	Wearing Glasses		296 (30%)	76 (30%)	76 (30%)	63 (25%)	81 (32%)
	Wearing Stethoscope		15 (2%)	4 (2%)	3 (1%)	5 (2%)	3 (1%)
	Wearing Scrubs		213 (21%)	56 (22%)	57 (23%)	55 (22%)	45 (18%)
	Wearing White Coat		534 (53%)	137 (55%)	124 (50%)	137 (55%)	136 (54%)
Comprehensive Ophthalmology (n = 100)	Men		63 (63%)	16 (64%)	15 (60%)	17 (68%)	15 (60%)
	Age ≥50 years		34 (34%)	8 (32%)	10 (40%)	8 (32%)	8 (32%)
	Race	White	78 (78%)	21 (84%)	19 (76%)	17 (68%)	21 (84%)
		Asian	10 (10%)	3 (12%)	4 (16%)	1 (4%)	2 (8%)
		Black	11 (11%)	1 (4%)	2 (8%)	6 (24%)	2 (8%)
		Latino	1 (1%)	0 (0%)	0 (0%)	1 (4%)	0 (0%)
	Wearing Glasses		24 (24%)	8 (32%)	6 (24%)	3 (12%)	7 (28%)
	Wearing Stethoscope		0 (0%)	0 (0%)	0 (0%)	0 (0%)	0 (0%)
	Wearing Scrubs		15 (15%)	3 (12%)	5 (20%)	2 (8%)	5 (20%)
	Wearing White Coat		64 (64%)	17 (68%)	13 (52%)	21 (84%)	13 (52%)
Cornea and External Disease (n = 100)	Men		71 (71%)	16 (64%)	17 (68%)	19 (76%)	19 (76%)
	Age ≥50 years		34 (34%)	11 (44%)	10 (40%)	5 (20%)	8 (32%)
	Race	White	71 (71%)	19 (76%)	20 (80%)	15 (60%)	17 (68%)
		Asian	26 (26%)	6 (24%)	5 (20%)	7 (28%)	8 (32%)
		Black	3 (3%)	0 (0%)	0 (0%)	3 (12%)	0 (0%)
		Latino	0 (0%)	0 (0%)	0 (0%)	0 (0%)	0 (0%)
	Wearing Glasses		25 (25%)	8 (32%)	7 (28%)	3 (12%)	7 (28%)
	Wearing Stethoscope		0 (0%)	0 (0%)	0 (0%)	0 (0%)	0 (0%)
	Wearing Scrubs		25 (25%)	7 (28%)	9 (36%)	1 (4%)	8 (32%)
	Wearing White Coat		61 (61%)	16 (64%)	14 (56%)	15 (60%)	16 (64%)
Glaucoma (n = 100)	Men		70 (70%)	18 (72%)	17 (68%)	18 (72%)	17 (68%)
	Age ≥50 years		24 (24%)	7 (28%)	6 (24%)	5 (20%)	6 (24%)
	Race	White	69 (69%)	19 (76%)	16 (64%)	17 (68%)	17 (68%)
		Asian	21 (21%)	4 (16%)	6 (24%)	5 (20%)	6 (24%)
		Black	10 (10%)	2 (8%)	3 (12%)	3 (12%)	2 (8%)
		Latino	0 (0%)	0 (0%)	0 (0%)	0 (0%)	0 (0%)
	Wearing Glasses		8 (8%)	1 (4%)	1 (4%)	4 (16%)	2 (8%)
	Wearing Stethoscope		0 (0%)	0 (0%)	0 (0%)	0 (0%)	0 (0%)
	Wearing Scrubs		24 (24%)	7 (28%)	6 (24%)	5 (20%)	6 (24%)
	Wearing White Coat		53 (53%)	11 (44%)	13 (52%)	15 (60%)	14 (56%)
Neuro-Ophthalmology (n = 100)	Men		57 (57%)	15 (60%)	16 (64%)	11 (44%)	15 (60%)
	Age ≥50 years		25 (25%)	3 (12%)	7 (28%)	7 (28%)	8 (32%)
	Race	White	74 (74%)	19 (76%)	18 (72%)	18 (72%)	19 (76%)
		Asian	17 (17%)	4 (16%)	5 (20%)	3 (12%)	5 (20%)
		Black	7 (7%)	2 (8%)	1 (4%)	3 (12%)	1 (4%)
		Latino	2 (2%)	0 (0%)	1 (4%)	1 (4%)	0 (0%)
	Wearing Glasses		26 (26%)	5 (20%)	7 (28%)	7 (28%)	7 (28%)
	Wearing Stethoscope		4 (4%)	1 (4%)	0 (0%)	3 (12%)	0 (0%)
	Wearing Scrubs		1 (1%)	0 (0%)	0 (0%)	1 (4%)	0 (0%)

Table S2: Ophthalmologist Characteristics Stratified by Subspecialty and Search Engine

	Wearing White Coat		65 (65%)	19 (76%)	12 (48%)	19 (76%)	15 (60%)
Ophthalmic Pathology (n = 100)	Men		67 (67%)	14 (56%)	14 (56%)	21 (84%)	18 (72%)
	Age ≥50 years		21 (21%)	4 (16%)	4 (16%)	7 (28%)	6 (24%)
	Race	White	77 (77%)	20 (80%)	19 (76%)	20 (80%)	18 (72%)
		Asian	13 (13%)	3 (12%)	3 (12%)	3 (12%)	4 (16%)
		Black	10 (10%)	2 (8%)	3 (12%)	2 (8%)	3 (12%)
		Latino	0 (0%)	0 (0%)	0 (0%)	0 (0%)	0 (0%)
	Wearing Glasses		29 (29%)	8 (32%)	8 (32%)	4 (16%)	9 (36%)
	Wearing Stethoscope		0 (0%)	0 (0%)	0 (0%)	0 (0%)	0 (0%)
	Wearing Scrubs		24 (24%)	6 (24%)	7 (28%)	7 (28%)	4 (16%)
	Wearing White Coat		56 (56%)	14 (56%)	14 (56%)	10 (40%)	18 (72%)
Ophthalmic Plastic Surgery (n = 100)	Men		57 (57%)	16 (64%)	13 (52%)	15 (60%)	13 (52%)
	Age ≥50 years		19 (19%)	6 (24%)	6 (24%)	2 (8%)	5 (20%)
	Race	White	68 (68%)	19 (76%)	16 (64%)	16 (64%)	17 (68%)
		Asian	31 (31%)	6 (24%)	9 (36%)	8 (32%)	8 (32%)
		Black	1 (1%)	0 (0%)	0 (0%)	1 (4%)	0 (0%)
		Latino	0 (0%)	0 (0%)	0 (0%)	0 (0%)	0 (0%)
	Wearing Glasses		44 (44%)	10 (40%)	11 (44%)	12 (48%)	11 (44%)
	Wearing Stethoscope		0 (0%)	0 (0%)	0 (0%)	0 (0%)	0 (0%)
	Wearing Scrubs		45 (45%)	12 (48%)	12 (48%)	12 (48%)	9 (36%)
	Wearing White Coat		27 (27%)	8 (32%)	6 (24%)	7 (28%)	6 (24%)
Pediatric Ophthalmology (n = 100)	Men		42 (42%)	10 (40%)	8 (32%)	14 (56%)	10 (40%)
	Age ≥50 years		20 (20%)	4 (16%)	3 (12%)	9 (36%)	4 (16%)
	Race	White	81 (81%)	19 (76%)	20 (80%)	21 (84%)	21 (84%)
		Asian	17 (17%)	6 (24%)	5 (20%)	2 (8%)	4 (16%)
		Black	1 (1%)	0 (0%)	0 (0%)	1 (4%)	0 (0%)
		Latino	1 (1%)	0 (0%)	0 (0%)	1 (4%)	0 (0%)
	Wearing Glasses		34 (34%)	8 (32%)	8 (32%)	8 (32%)	10 (40%)
	Wearing Stethoscope		4 (4%)	2 (8%)	1 (4%)	0 (0%)	1 (4%)
	Wearing Scrubs		6 (6%)	2 (8%)	2 (8%)	2 (8%)	0 (0%)
	Wearing White Coat		74 (74%)	19 (76%)	17 (68%)	21 (84%)	17 (68%)
Low-Vision (n = 100)	Men		58 (58%)	14 (56%)	17 (68%)	12 (48%)	15 (60%)
	Age ≥50 years		47 (47%)	14 (56%)	14 (56%)	7 (28%)	12 (48%)
	Race	White	78 (78%)	20 (80%)	20 (80%)	19 (76%)	19 (76%)
		Asian	11 (11%)	2 (8%)	3 (12%)	4 (16%)	2 (8%)
		Black	10 (10%)	3 (12%)	2 (8%)	1 (4%)	4 (16%)
		Latino	1 (1%)	0 (0%)	0 (0%)	1 (4%)	0 (0%)
	Wearing Glasses		40 (40%)	9 (36%)	10 (40%)	13 (52%)	8 (32%)
	Wearing Stethoscope		5 (5%)	1 (4%)	2 (8%)	0 (0%)	2 (8%)
	Wearing Scrubs		12 (12%)	1 (4%)	3 (12%)	5 (20%)	3 (12%)
	Wearing White Coat		43 (43%)	12 (48%)	12 (48%)	8 (32%)	11 (44%)
Medical Retina (n = 100)	Men		75 (75%)	18 (72%)	19 (76%)	16 (64%)	22 (88%)
	Age ≥50 years		23 (23%)	4 (16%)	4 (16%)	11 (44%)	4 (16%)
	Race	White	74 (74%)	20 (80%)	21 (84%)	14 (56%)	19 (76%)
		Asian	22 (22%)	5 (20%)	4 (16%)	7 (28%)	6 (24%)
		Black	3 (3%)	0 (0%)	0 (0%)	3 (12%)	0 (0%)
		Latino	1 (1%)	0 (0%)	0 (0%)	1 (4%)	0 (0%)
	Wearing Glasses		35 (35%)	10 (40%)	9 (36%)	5 (20%)	11 (44%)
	Wearing Stethoscope		2 (2%)	0 (0%)	0 (0%)	2 (8%)	0 (0%)
	Wearing Scrubs		23 (23%)	6 (24%)	7 (28%)	6 (24%)	4 (16%)
	Wearing White Coat		50 (50%)	12 (48%)	12 (48%)	11 (44%)	15 (60%)
	Men		82 (82%)	20 (80%)	21 (84%)	19 (76%)	22 (88%)

Table S2: Ophthalmologist Characteristics Stratified by Subspecialty and Search Engine

Vitreoretinal Surgery (n = 100)	Age ≥50 years		28 (28%)	5 (20%)	9 (36%)	6 (24%)	8 (32%)
	Race	White	67 (67%)	17 (68%)	15 (60%)	19 (76%)	16 (64%)
		Asian	30 (30%)	8 (32%)	10 (40%)	3 (12%)	9 (36%)
		Black	3 (3%)	0 (0%)	0 (0%)	3 (12%)	0 (0%)
		Latino	0 (0%)	0 (0%)	0 (0%)	0 (0%)	0 (0%)
	Wearing Glasses		31 (31%)	9 (36%)	9 (36%)	4 (16%)	9 (36%)
	Wearing Stethoscope		0 (0%)	0 (0%)	0 (0%)	0 (0%)	0 (0%)
	Wearing Scrubs		38 (38%)	12 (48%)	6 (24%)	14 (56%)	6 (24%)
	Wearing White Coat		41 (41%)	9 (36%)	11 (44%)	10 (40%)	11 (44%)

This table presents the demographics by ophthalmic subspecialty and search engine used. Data are presented as frequency n (%). The "All Engines Combined" column represents the aggregate data across all four search engines (total n = 1000; n = 100 per subspecialty). The individual search engine columns (Bing, DuckDuckGo, Google, Yahoo!) represent the data specific to that platform (total n = 250; n = 25 per subspecialty). Within each subspecialty, each search engine contributes n = 25 images (total n = 100 per subspecialty across all four search engines). Percentages are calculated based on the number of images within that specific search engine and subspecialty intersection (i.e., within-cell denominator n = 25).
